# SWI/SNF complexes act through CBP-1 histone acetyltransferase to regulate acute functional tolerance to alcohol

**DOI:** 10.1186/s12864-020-07059-y

**Published:** 2020-09-21

**Authors:** Laura D. Mathies, Jonathan H. Lindsay, Amal P. Handal, GinaMari G. Blackwell, Andrew G. Davies, Jill C. Bettinger

**Affiliations:** Department of Pharmacology and Toxicology, Virginia Commonwealth University, PO Box 980613, Richmond, VA 23298 USA

**Keywords:** SWI/SNF chromatin remodeling, Ethanol, Alcohol, *C. elegans*, Transcriptome, *cbp-1*, Histone acetyltransferase, Differential gene expression

## Abstract

**Background:**

SWI/SNF chromatin remodeling genes are required for normal acute responses to alcohol in *C. elegans* and are associated with alcohol use disorder in two human populations. In an effort to discover the downstream genes that are mediating this effect, we identified SWI/SNF-regulated genes in *C. elegans*.

**Results:**

To identify SWI/SNF-regulated genes in adults, we compared mRNA expression in wild type and *swsn-1(os22ts)* worms under conditions that produce inactive *swsn-1* in mature cells. To identify SWI/SNF-regulated genes in neurons, we compared gene expression in *swsn-9(ok1354)* null mutant worms that harbor a neuronal rescue or a control construct*.* RNA sequencing was performed to an average depth of 25 million reads per sample using 50-base, paired-end reads. We found that 6813 transcripts were significantly differentially expressed between *swsn-1(os22ts)* mutants and wild-type worms and 2412 transcripts were significantly differentially expressed between *swsn-9(ok1354)* mutants and *swsn-9(ok1354)* mutants with neuronal rescue. We examined the intersection between these two datasets and identified 603 genes that were differentially expressed in the same direction in both comparisons; we defined these as SWI/SNF-regulated genes in neurons and in adults. Among the differentially expressed genes was *cbp-1,* a *C. elegans* homolog of the mammalian CBP/p300 family of histone acetyltransferases. CBP has been implicated in the epigenetic regulation in response to alcohol in animal models and a polymorphism in the human CBP gene, CREBBP, has been associated with alcohol-related phenotypes. We found that *cbp-1* is required for the development of acute functional tolerance to alcohol in *C. elegans*.

**Conclusions:**

We identified 603 transcripts that were regulated by two different SWI/SNF complex subunits in adults and in neurons. The SWI/SNF-regulated genes were highly enriched for genes involved in membrane rafts, suggesting an important role for this membrane microdomain in the acute alcohol response. Among the differentially expressed genes was *cbp-1;* CBP-1 homologs have been implicated in alcohol responses across phyla and we found that *C. elegans cbp-1* was required for the acute alcohol response in worms.

## Background

Alcohol is a widely used and abused drug. The significant majority of adults in the United States uses alcohol, and the misuse of alcohol has serious societal impacts [1]. Disturbingly, problems associated with alcohol misuse appear to be getting worse; for example, the rate of deaths associated with alcohol use has risen substantially in the last two decades, particularly among groups that have traditionally lagged in alcohol-related mortality [2].

The liability to abuse alcohol is strongly influenced by an individual’s genetics [3], although the specific genes involved are as yet incompletely understood. An individual’s level of response (LR) to alcohol manifests earlier than alcohol problems, and is a strong predictor of the likelihood to develop alcohol use disorder (AUD) [4–6]. LR to alcohol is a composite assessment that consists of both physiological measures, such as body sway, as well as subjective measures of intoxication. A lower sensitivity to the depressing effects of alcohol early in adulthood is correlated with a higher lifetime incidence of alcohol problems [7–9]. Identifying the genes that modulate the LR to alcohol is the goal of our work.

We use the nematode *Caenorhabditis elegans* as a model for understanding the acute physiological response to alcohol (ethanol), and for identifying genes that are likely to affect LR in humans. We previously found that SWI/SNF chromatin remodeling complex genes were required for normal acute ethanol response behaviors in *C. elegans* [10]. SWI/SNF are multi-protein complexes that modify chromatin structure by regulating the interaction of histones and DNA at particular genes, occluding or revealing transcription factor binding sites (reviewed in [11]); these actions result in an epigenetic modulation of target gene expression. SWI/SNF complexes share common enzymatic core proteins, and different complexes have different accessory subunits that confer their sequence specificity (reviewed in [12, 13]). In worms, most SWI/SNF complex members were involved in regulating acute ethanol response behaviors, but different SWI/SNF complexes affected different behaviors: BAF subunits were required for normal initial sensitivity to ethanol, while PBAF subunits were required for acute functional tolerance to ethanol [10]. We found that allelic variation in several SWI/SNF complex members was associated with alcohol dependence in different adult human populations [10, 14]. Allelic variation in different SWI/SNF complex members was also associated with antisocial behavior in adolescents, a phenotype that is strongly predictive of future alcohol use problems [14].

Together, these data strongly argue that the SWI/SNF complex plays a functional role in human AUD, and support the investigation of the biological underpinnings of this role. Because SWI/SNF acts to regulate transcription, the downstream effectors through which it modulates the acute ethanol response are of significant interest. Here, we use transcriptional profiling in *C. elegans* to identify these downstream modulators. We took advantage of two genetic reagents, one that localizes expression of *swsn-9* to neurons using tissue-specific rescue of a *swsn-9* null allele, and one that temporally restricts function of *swsn-1* to adults using a temperature-sensitive allele of *swsn-1* [15]. *swsn-9* encodes a PBAF subunit, while *swsn-1* encodes a core subunit that is predicted to be present in both BAF and PBAF; both genes are required for acute functional tolerance to ethanol [10]. We found that 603 genes were regulated by the SWI/SNF complex in adults and in neurons. Among these genes was *cbp-1*, encoding a histone acetyltransferase. We found that animals carrying loss of function alleles of *cbp-1* had defects in acute ethanol response behaviors, indicating that *cbp-1* is an important SWI/SNF target for LR to ethanol.

## Results

### SWI/SNF-regulated genes in adults

**Fig. 1:**
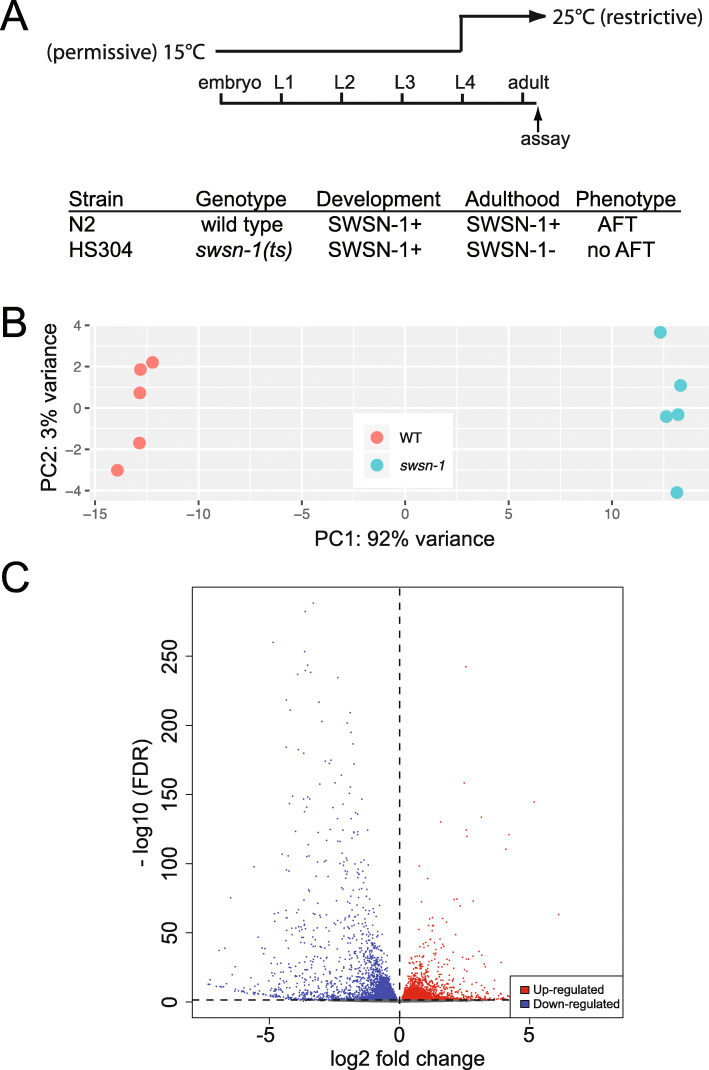
SWI/SNF-regulated genes in adults. **a** A temperature-sensitive allele, *swsn-1(os22ts),* was used to demonstrate that SWI/SNF complexes are required in adult tissues for AFT [10]. Diagram (top) shows the temperature shift regimen and table (bottom) summarizes alcohol response behaviors from this experiment. **b** Principal component analysis with gene expression plotted relative to the first two principal components (PC1 and PC2). Wild type and *swsn-1(os22ts)* replicates are most similar to replicates of the same genotype. **c** Volcano plot shows genes that are up-regulated (red) and down-regulated (blue) in *swsn-1(os22ts)* mutants. Dashed lines indicate the FDR and fold change cutoffs (FDR ≤ 0.05 and fold change ≥1)

We previously showed that *swsn-1* is required during adulthood for the development of acute functional tolerance (AFT) to ethanol [10]. *swsn-1(os22ts)* is a temperature sensitive allele of *swsn-1*; *os22ts* is an amino acid substitution that causes the protein encoded by *swsn-1(os22ts)* to be functional at 15 °C, but destabilized so that its function is lost at 25 °C [15]. For these experiments, we reared N2 and *swsn-1(os22ts)* at the permissive temperature (15 °C) from the time the eggs were laid through the fourth larval stage (L4), at which time we shifted the worms to the restrictive temperature (25 °C) and grew them to adulthood (Fig. 1a). In wild type worms, SWSN-1 is expressed both during development and in adulthood. In *swsn-1(os22ts)* mutant worms, this temperature shift protocol produces functional SWSN-1 during development and inactive SWSN-1 in adulthood. We found that worms lacking SWSN-1 during adulthood failed to develop AFT, while wild type worms reared under the same conditions developed AFT to ethanol [10]. This result suggests that the SWI/SNF complex regulates gene expression in adults that is necessary for AFT in these animals. To identify genes that are regulated by SWSN-1 in adults, we performed RNA sequencing on wild type and *swsn-1(os22ts)* mutant worms that were subjected to the temperature shift protocol. Five biological replicates were generated on different days; mutant and wild type worms were reared in parallel, thus limiting the effect of environmental variation on our comparison. We isolated RNA from young adult worms approximately 6 h after the temperature shift, and at a time when they were beginning to lay eggs. RNA sequencing libraries were prepared and sequenced by the Genomic Services Laboratory at Hudson Alpha (Huntsville, AL).

We assessed the correlation between biological replicates using principal component analysis and found that our biological replicates grouped together and the two genotypes cluster in distinct groups (Fig. 1b). The first two principal components accounted for 95% of the variance in the dataset, with principal component one (variation between sample types) accounting for 92% of the variance. We analyzed differential gene expression using DESeq2 [16] and found that 6813 genes were differentially expressed between *swsn-1(os22ts)* mutant and wild type worms (FDR ≤ 0.05) (Additional file 1). A volcano plot shows the wide distribution of differentially expressed genes (DEGs) (Fig. 1c). Approximately equal numbers of genes had increased (3210) and decreased (3603) expression in *swsn-1(os22ts)* mutants when compared to wild type. This suggests that the SWI/SNF complex both activates and represses gene expression in adult tissues, which is consistent with previous observations of SWI/SNF regulatory activity [17–20].

### SWI/SNF-regulated genes in neurons

We previously showed that *swsn-9(ok1354)* mutant worms were unable to develop AFT to ethanol and that, if we expressed *swsn-9::GFP* in all neurons under the control of the *ric-19* promoter, we could restore AFT to *swsn-9(ok1354)* mutants [10]. This result indicates that the SWI/SNF complex regulates gene expression in neurons that is important for AFT.

**Fig. 2:**
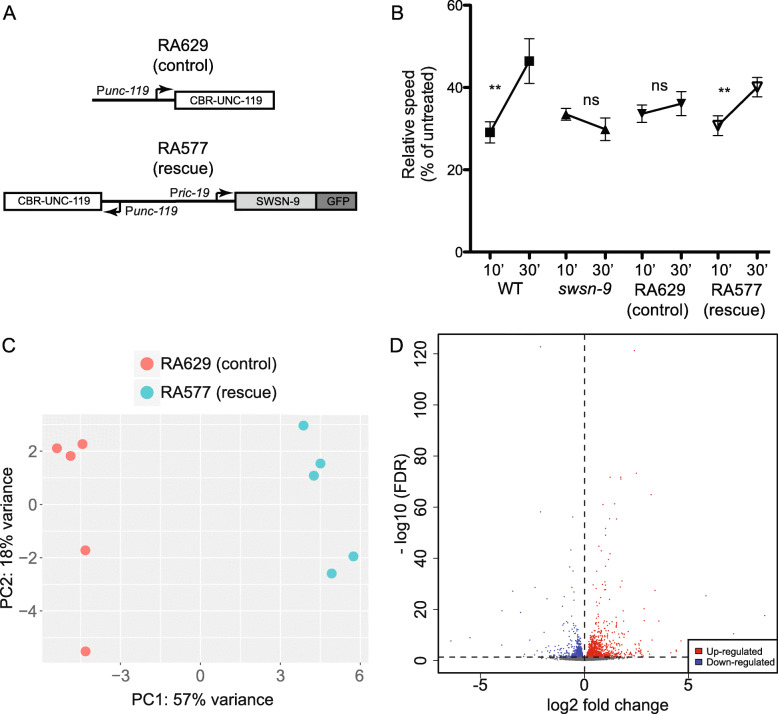
SWI/SNF-regulated genes in neurons. **a** Constructs for single-copy rescue of *swsn-9(ok1354)* mutants in neurons. The empty MosSCI vector (top) serves as a negative control and contains the *unc-119+* selectable marker*.* The neuronal rescue plasmid (bottom) expresses *SWSN-9::GFP* under control of the *ric-19* promoter; it also contains the *unc-119+* selectable marker. **b** Animals were tested on 0 mM and 400 mM exogenous ethanol. Relative speeds were calculated as treated over untreated speeds. The development of AFT is indicated by a statistically significant recovery of speed between 10 and 30 min of exposure. All genotypes on the same graph were tested simultaneously on the same plates. Wild-type (WT) animals develop AFT, while *swsn-9(ok1354)* mutants fail to develop AFT (*n* = 6). A single copy insertion of *Pric-19::SWSN-9::GFP* (rescue) was able to rescue the AFT defect of *swsn-9(ok1354)* mutants, while a single copy insertion of the empty MosSCI vector (control) did not rescue the AFT defect. Error bars indicate S.E.M. Paired one-tailed Student’s *t* tests were used for statistical comparisons of speeds at 10 and 30 min. One-way ANOVA, with Tukey’s multiple comparison test, was used to compare initial sensitivity across the strains; no significant difference was observed. For indicated comparisons: ns, not significant; **p ≤ 0.01 **c** Principal component analysis with gene expression plotted relative to the first two principal components (PC1 and PC2). *swsn-9(ok1354)* mutant replicates containing the rescue or control constructs are most similar to replicates of the same genotype. **d** Volcano plot shows genes that are up-regulated (red) and down-regulated (blue) in *swsn-9(ok1354)* mutant neurons. Dashed lines indicate the FDR and fold change cutoffs (FDR ≤ 0.05 and fold change ≥1)

Our previous experiments used extra-chromosomal arrays to show that *swsn-9* was required in neurons. Extra-chromosomal arrays are multicopy DNA concatamers that are transmitted with varying frequency through mitosis and meiosis [21]. In order to have uniform transgene expression and normal diploid gene dosage, we generated single copy insertions of the neuronal expression construct *Pric-19::swsn-9::GFP* using the Mos-1 mediated single copy insertion (MosSCI) technique [22]. The MosSCI targeting vector contains a selectable marker; therefore we also generated single copy insertions of the empty vector as a control (Fig. 2a). We crossed these insertions into *swsn-9(ok1354)* and tested them for rescue of the *swsn-9(ok1354)* AFT defect (Fig. 2b). Wild type worms develop AFT, while *swsn-9(ok1354)* worms fail to develop AFT. We found that single copy insertions of the empty vector did not rescue AFT, while single copy insertions containing *Pric-19::swsn-9::GFP* did rescue the AFT defect of *swsn-9(ok1354)* null mutants.

In order to identify SWI/SNF-regulated neuronal genes, we performed RNA sequencing on *swsn-9(ok1354)* mutants harboring either the empty vector control or the neuronal *swsn-9::GFP* rescue construct. We collected five biological replicates on different days and rescue and control worms were reared in parallel. We isolated total RNA from young adult worms of each genotype. RNA sequencing libraries were prepared and sequenced by the Genomic Services Laboratory at Hudson Alpha (Huntsville, AL).

We assessed the correlation between biological replicates using principal component analysis and found that our biological replicates grouped together (Fig. 2c). The first two principal components accounted for 75% of the variance in the dataset, with principal component one (variation between sample types) accounting for 57% of the variance. We analyzed differential gene expression using DESeq2 [16] and found that 2412 genes were differentially expressed between *swsn-9(ok1354)* mutants with and without neuronal rescue (FDR ≤ 0.05) (Additional file 1). A volcano plot shows the distribution of DEGs (Fig. 2d). Consistent with our *swsn-1(os22ts)* data, we found that similar numbers of genes were down-regulated (1157) as were up-regulated (1255).

### SWI/SNF-regulated genes, in neurons, and in adults

**Fig. 3:**
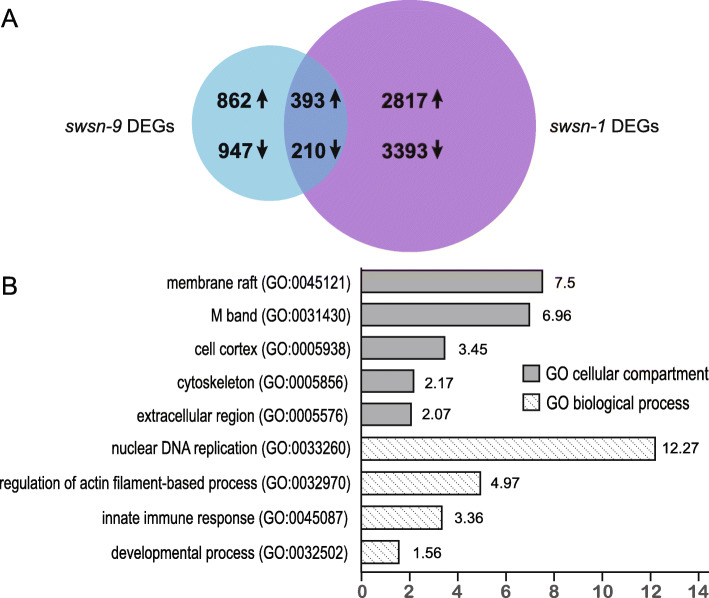
SWI/SNF-regulated genes in neurons and adults. **a** Differential gene expression analysis identified 6813 genes with differential expression between *swsn-1(os22ts)* and wild type adult worms (purple) and 2412 genes between *swsn-9(ok1354)* mutants with and without neuronal rescue (blue). Of the 603 DEGs that were regulated in the same direction in both comparisons, 392 were up-regulated, and 210 were down-regulated. **b** PANTHER GO biological process and cellular compartment terms enriched in this gene set; there were no significant GO molecular function terms. GO terms are plotted against the fold enrichment relative to the expected number of genes in lists of these sizes

The intersection of these two datasets identifies genes that are differentially expressed in neurons and in adults, in SWI/SNF mutants relative to wild type. Because we isolated mRNA from whole adult animals and we expect that the relevant gene expression changes will be in a small number of cells, we have not used any fold-change cutoff for this analysis. We identified 603 genes that were differentially expressed in the same direction in both *swsn-1(os22ts)* and *swsn-9(ok1354)* mutants compared to wild type (FDR ≤ 0.05); 393 genes were up-regulated and 210 were down-regulated (Fig. 3a). These 603 genes are excellent candidates for mediators of the SWI/SNF effect on AFT.

### GO term enrichment analysis

We performed an analysis to determine if particular functional classes of genes are overrepresented in our DEGs. We found that gene ontology (GO) biological process terms associated with metabolic processes, such as “mitochondrial ATP synthesis coupled electron transport” (GO:0042775) and “carboxylic acid catabolic process” (GO:0046395), were overrepresented in the *swsn-1(os22ts)* DEGs (Additional file 2). By contrast, we found a wide range of overrepresented GO biological process terms for the *swsn-9(ok1354)* DEGs, ranging from “regulation of cell shape” (GO:0008360) to “translation initiation” (GO:0006413) to “mitochondrial gene expression” (GO:0140053) (Additional file 2). Interestingly, we found that the GO biological process term “fatty acid metabolic process” (GO:0006631) was overrepresented in both the *swsn-1(os22ts)* and *swsn-9(ok1354)* DEGs, with 2.4 fold enrichment in the *swsn-9(ok1354)* DEG set and 1.8 fold enrichment in the *swsn-1(os22ts)* DEGs. Finally, we performed gene ontology GO term overrepresentation tests for genes at the intersection of our two datasets (Additional file 2). We found 7.5 times as many genes with the GO cellular process term “membrane rafts” (GO:0045121) and 3.4 times as many genes with the GO biological process term “innate immune response” (GO:0045087) as would be expected for a gene list of this size (Fig. 3b).

### Comparison of *swsn-1* and *swsn-9* regulated genes

**Fig. 4:**
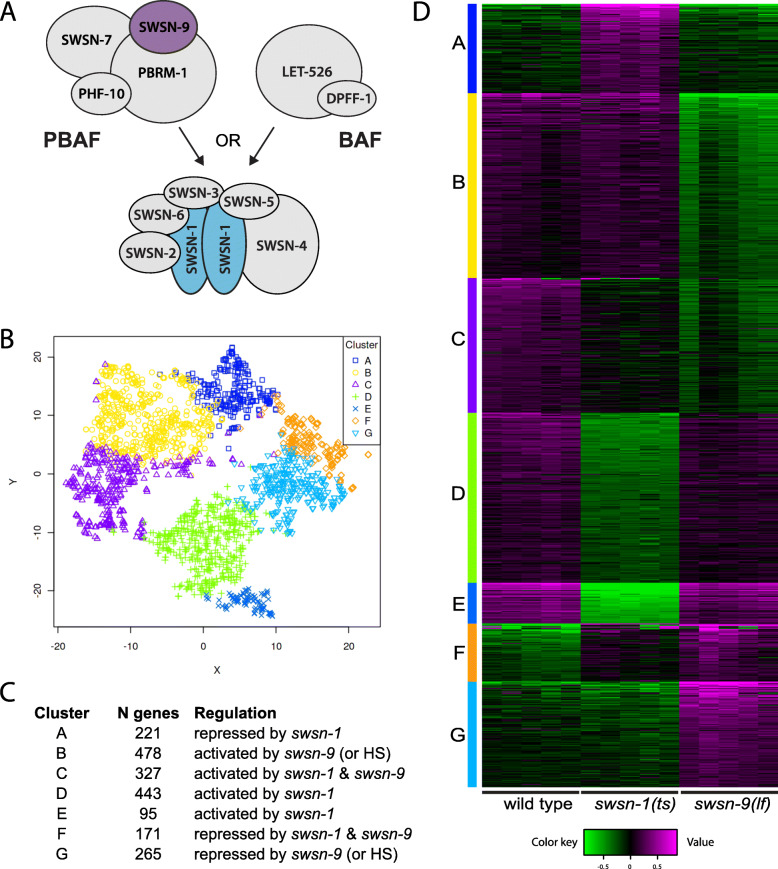
Comparison of *swsn-1* and *swsn-9* gene expression. **a** Diagram of SWI/SNF complexes in *C. elegans.* Genetic studies support the presence of distinct BAF and PBAF complexes that form by the association of unique subunits with the core complex. The SWSN-1 (blue) and SWSN-9 (purple) proteins are indicated. **b-d** k-means clustering of the top 2000 most variable genes across three genotypes (five replicates per genotype). **b** t-SNE map shows distinct clustering for seven k-means clusters. **c** Description of the seven clusters. **d** Heatmap of clustered genes; genotypes are indicated on the bottom

SWI/SNF complexes are composed of multiple subunits that combine to form functionally distinct complexes [reviewed in [12, 13]]. Our two RNA sequencing datasets utilized different SWI/SNF mutants: *swsn-1* encodes a core subunit that is present in all SWI/SNF complexes, while *swsn-9* encodes an accessory subunit that is specific to the PBAF complex in worms (Fig. 4a) [10, 23]. We have an opportunity with these datasets to compare the effect of loss of different SWI/SNF subunits on gene expression. We examined the 2000 most variable genes in wild type, *swsn-1(os22ts),* and *swsn-9(ok1354)* using k-means clustering (Fig. 4b-d; Additional file 3). As expected, we identified clusters containing genes that were up- or down-regulated in *swsn-1(os22ts)* (clusters A, D, and E) and *swsn-9(ok1354)* mutants (clusters B and G). Note that clusters B and G contain genes that are regulated by *swsn-9* and also genes that respond to temperature, since the wild type and *swsn-1(os22ts)* worms were shifted from 15 °C to 25 °C prior to gene expression analysis, whereas *swsn-9(ok1354)* mutants were not. We also identified clusters of genes that are regulated similarly in both *swsn-1(os22ts)* and *swsn-9(ok1354)* mutants (clusters C and F). These genes are regulated by the PBAF complex in adult neurons. Cluster C contains genes that are normally activated, while Cluster F contains genes that are normally repressed by PBAF.

We asked if our PBAF target genes were regulated by PBAF in other systems. Chowdhury et al. identified 2464 genes that were differentially expressed in a renal cell carcinoma model upon re-expression of PBRM-1 [24]. We identified human homologs of the genes in clusters C and F using the DRSC Integrative Ortholog Prediction Tool (DIOPT) [25], retaining any genes with a DIOPT score greater than two. This resulted in lists of 336 and 312 unique human homologs of the genes in clusters C and F, respectively. We compared these lists of human genes with the DEGs found in the Chowdhury study and found that 11.2% of the cluster C and F homologs were regulated by PBRM-1 in cancer cell lines (Additional file 3).

### Human homologs of SWI/SNF-regulated genes are associated with alcohol-related phenotypes

We are interested in finding SWI/SNF-regulated genes that are mediating the effects of the SWI/SNF complex on human alcohol-related phenotypes. We identified human homologs of the 603 worm genes identified at the intersection of our two datasets using DIOPT [25]. This resulted in a list of 874 unique human genes. We generated a list of human genes for which there was a reported gene disease association in the literature using DisGenNet [26]. We included disease terms related to alcohol or substance misuse, consumption, and withdrawal. Among the 874 human homologs of our SWI/SNF-regulated genes we found that 84 (9.6%) are linked to one or more alcohol or substance use phenotypes in the DisGeNET database (Additional file 4). Next, we used the human gene list to query PubMed for publications linking these genes to problematic alcohol use (search terms include “alcoholism”, “alcohol use disorder”, or “alcohol dependence”) or to alcohol more broadly (“alcohol” or “ethanol”). We found 418 genes with one or more alcohol publications and 224 genes with one or more alcohol phenotype publications (Additional file 4). We sought to identify epigenetic regulators among our SWI/SNF-regulated genes because epigenetic regulators, like SWI/SNF, often work together. We queried PubMed for any citations linking these genes to epigenetic regulation and found 518 genes with one or more PubMed citations containing the search term “epigenetic”. Finally, we generated a list of 195 genes for which there is literature evidence for both alcohol misuse phenotypes and epigenetic mechanisms of action (Additional file 4). These 195 genes were ranked based on the number of citations linking the genes to epigenetics; the top twenty genes are reported in Table 1. These are our top candidate genes for future study.

**Table 1:** Top 20 human genes linked to alcohol problems and epigenetics

Human gene	PubMed Citations		Worm Gene Expression	
	Alcohol problems	Epigenetics	Gene	Expression
STAT3	87	363	*sta-1*	Up
GSTP1	19	284	*gst-16*	Down
			*gst-39*	Up
			*gst-38*	Up
			*gst-19*	Down
EP300	1	153	*cbp-1*	Down
NOTCH1	7	149	*glp-1*	Down
SLC6A4	193	123	*snf-11*	Up
CREBBP	6	123	*cbp-1*	Down
STAT1	26	113	*sta-1*	Up
TLR4	202	102	*tol-1*	Down
SFN	2	96	*ftt-2*	Up
VDR	7	95	*nhr-8*	Up
RHOA	7	62	*rho-1*	Down
TLR2	42	58	*tol-1*	Down
FRY	19	58	*sax-2*	Down
ABCB1	6	58	*pgp-5*	Up
AGO2	2	58	*alg-2*	Down
			*alg-5*	Down
HNF4A	9	56	*nhr-35*	Up
			*nhr-60*	Down
STAT4	4	45	*sta-1*	Up
RAC1	6	42	*ced-10*	Down
LMNA	2	41	*ifd-2*	Down
			*ifd-1*	Up
KAT5	1	40	*mys-4*	Down

### The histone acetyltransferase CBP-1 is dose-dependently required for AFT

**Fig. 5:**
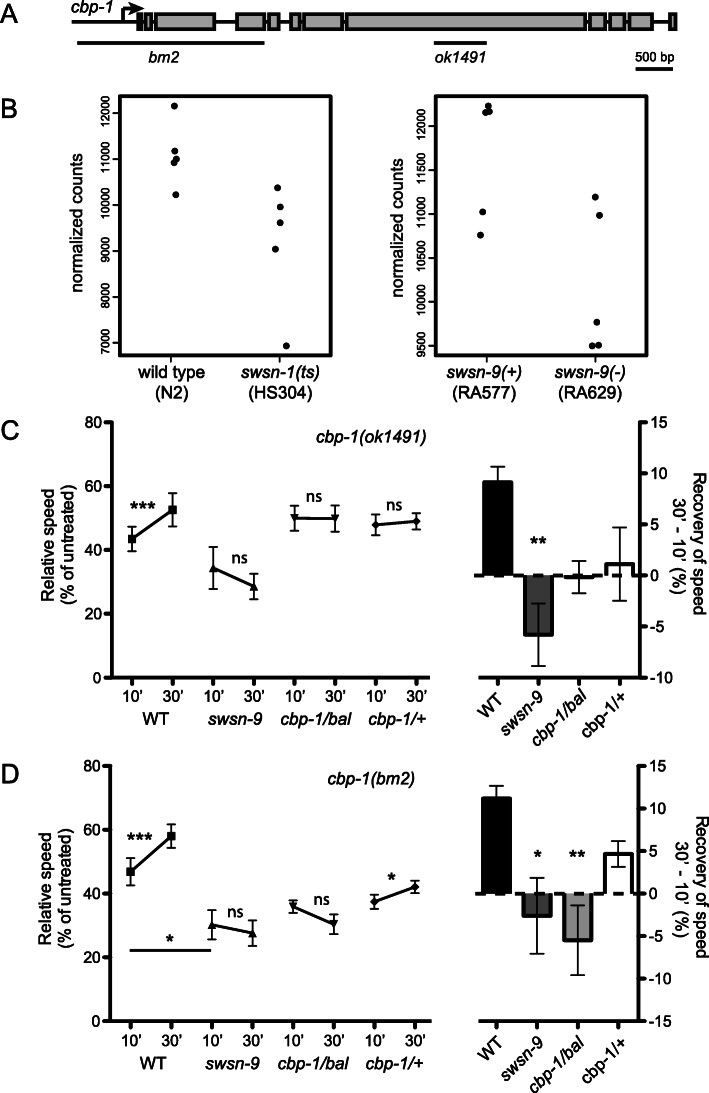
*cbp-1* is required for AFT. **a** A model of the *cbp-1* gene indicating exon/intron structure and the location and extent of the deletion alleles used in this study. **b** Normalized *cbp-1* expression in our RNA sequencing samples. **c-d** Animals were tested on 0 mM and 400 mM exogenous ethanol. Relative speeds were calculated as treated over untreated speeds (left Y-axes). AFT is quantified as the recovery of speed between 10 and 30 min of exposure (right Y-axes). All genotypes on the same graph were tested simultaneously on the same plates. Wild-type (N2) animals develop AFT; *swsn-9(ok1354)* mutant animals do not develop AFT. **c** Heterozygous *cbp-1(ok1491)* mutant animals over either a balancer (bal) or wild type (+) chromosome failed to develop AFT. **d** Heterozygous *cbp-1(bm2)* mutant animals maintained over a balancer (bal) chromosome failed to develop AFT, while heterozygous *cbp-1(bm2)* over a wild type chromosome did develop AFT; the recovery of speed was statistically different from wild-type for *cbp-1(bm2)/balancer*. Error bars indicate S.E.M. Paired one-tailed Student’s *t* tests were used for statistical comparisons of speeds at 10 and 30 min; One-way ANOVA, with Tukey’s multiple comparison test, was used to compare the initial sensitivity and recovery of speed across the strains. For indicated comparisons: ns, not significant; *p ≤ 0.05; **p ≤ 0.01; ***p ≤ 0.001

Among our top candidate genes is the *C. elegans* gene encoding CBP, *cbp-1*, which is similarly homologous to two closely related human genes, CREBBP and EP300. Both CREBBP and EP300 appear in our top 20 genes related to human alcohol problems and epigenetics (Table 1), strongly supporting our interest in *cpb-1*. CBP homologs have well described roles in the nervous system response to alcohol in animal models [27–31], and CREBBP is associated with addiction in humans [32]. We reasoned that if SWI/SNF is regulating ethanol response behaviors in part through its regulation of *cbp-1*, then disrupting function of *cbp-1* itself should alter ethanol response behaviors. *cbp-1* expression was decreased relative to wild-type in *swsn-1(os22ts)* and *swsn-9(ok1354)* mutants (Fig. 5b), both of which are defective in acute functional tolerance (AFT), so we predicted that a loss of *cbp-1* should decrease or eliminate AFT. We therefore tested two different strong loss-of-function alleles of *cbp-1* in our behavioral assays (Fig. 5a). Mutants carrying homozygous *cbp-1* loss-of-function alleles die, so we tested *cbp-1* heterozygotes. We found that *cbp-1(ok1491)* heterozygotes did not develop AFT (Fig. 5c). We found that *cbp-1(bm2)/balancer* heterozygotes had reduced AFT, whereas *cbp-1(bm2)/+* heterozygyotes had no AFT to ethanol (Fig. 5d). This supports a model in which SWI/SNF acts, at least in part, through its regulation of *cpb-1* in ethanol response behaviors.

## Discussion

The SWI/SNF chromatin remodeling complex is required in *C. elegans* for the development of acute functional tolerance to ethanol [10]. In this study, we identified the set of transcripts that are regulated by the SWI/SNF chromatin remodeling complex in adult neurons. To identify the transcripts under the regulatory control of *swsn-9* in neurons, we generated a strain in which we provided *swsn-9* expression only in neurons in an otherwise *swsn-9(ok1354)* mutant background (Fig. 1). By comparing the transcriptome of these animals to that of *swsn-9(ok1354)* mutant animals, we could find transcripts regulated by the SWI/SNF complex in neurons. We found that 2412 genes were differentially expressed in this sample, suggesting that these genes are under the control of a SWSN-9 containing SWI/SNF complex in neurons. To identify transcripts that are under the regulatory control of *swsn-1* in adults, we used the special temperature sensitive allele *swsn-1(os22ts)* to provide SWSN-1 function throughout development, then eliminate SWSN-1 function in adults (Fig. 2). We compared the transcriptomes of animals with and without SWSN-1 function in adulthood to identifiy candidate AFT mediators. We found that 6813 genes are under regulatory control by *swsn-1* in adults. These effects on gene expression may be direct or indirect; for example, some may be the result of SWI/SNF’s regulation of one or more transcriptional repressors or activators that themselves regulate expression of the genes.

We observed that more than two times as many genes were regulated by *swsn-1* than by *swsn-9* in our study; this was consistent with our expectations because SWSN-9 is an accessory subunit that is predicted to be found only in PBAF complexes, while SWSN-1 is a core subunit that is predicted to be present in both BAF and PBAF complexes. Further, this difference in the number of regulated genes may also be due in part to the fact that our *swsn-9(ok1354)* dataset is confined to neuronally expressed genes, whereas the *swsn-1(os22ts)* dataset includes all tissues. Interestingly, in addition to different numbers of genes, we also found that the *swsn-1* and *swsn-9* datasets consisted of mostly non-overlapping sets of genes. If these two SWI/SNF complex subunits were acting together for most or all gene regulation by PBAF, then we would expect that the *swsn-9* neuronally-regulated dataset would largely be a subset of the *swsn-1*-pan tissue regulated dataset. In fact, we observed the contrary; most of the genes in each dataset do not appear in the other dataset.

There are several possible explanations for this lack of regulatory overlap. One possibility is that, in addition to acting together in some cases, these two subunits also participate in separate complexes that target different genes. This would imply that SWSN-9 can act with other as yet undetermined proteins to regulate transcription independent of its known function with SWSN-1. This would be a non-canonical function of SWSN-9, and while our data do not exclude this possibility, we do not favor this hypothesis. A second possibility is that *swsn-1(os22ts)* may be non-null at 25 °C, so that it does provide some function in our restrictive conditions. In such a case, we would see DEGs disrupted by the *swsn-9(ok1354)* null mutation that are not disrupted by *swsn-1(os22ts)* because of its residual activity, but those genes nevertheless do require the function of SWSN-1. We favor a third explanation: in our experimental paradigm, *swsn-1(os22ts)* function is only disrupted in the adult, and its function is more normal during development. In contrast, *swsn-9(ok1354)* is non-functional throughout development. We hypothesize that the *swsn-9*-specific DEGs are regulated by SWI/SNF before adulthood. We do not observe their dysregulation by the *swsn-1(os22ts)* allele because in this work it functions normally during development. Thus, the *swsn-9*-specific DEGs represent developmental gene regulation defects that persist into adulthood. Our datasets provide a rich resource with which to examine the functions of these SWI/SNF target genes.

Although it remains possible that *swsn-1* and *swsn-9* regulate AFT through independent mechanisms, because we found that loss of either *swsn-9* or *swsn-1* function causes similar defects in AFT [10], we favor the idea that they act together in the SWI/SNF complex for AFT. Therefore, our primary interest was in genes that are regulated by both of these subunits. We found that a total of 603 genes were regulated in the same direction by both *swsn-9* and *swsn-1*. These are candidate AFT mediators. To begin to characterize these genes, we first looked at overrepresented classes of genes, with the goal of identifying biological processes that are likely to be involved in AFT.

### SWI/SNF regulates the membrane microenvironment

We were particularly intrigued by our observation that the most overrepresented category in the intersection of the *swsn-9* and *swsn-1* regulated genes are those annotated to be involved in membrane rafts, which are a form of membrane microarchitecture characterized by particular lipid comoposition, thick bilayer and the presence of cholesterol. Membrane microachitecture can provide a platform for regulating the activity of many membrane proteins, both directly and indirectly. For example, membrane characteristics such as lipid makeup, bilayer thickness, and cholesterol content have all been shown to modulate the activity of the BK channel (reviewed in [33, 34]), which is a well characterized ethanol target (reviewed in [35, 36]). In addition, membrane microdomains such as lipid rafts can regulate the access of proteins to effector molecules, thereby affecting their activity [37, 38]. Importantly, we have previously demonstrated that cholesterol, a key component of lipid rafts, is required for the development of AFT [39]. In addition, we have shown that levels of the long chain ω-3 polyunsaturated fatty acid, eicosapentaneoic acid (EPA), tune the levels of AFT; EPA is a component of lipid rafts [40, 41]. Taken together, these data point to a probable role for rafts in AFT. Our gene expression data implicate SWI/SNF complexes in the regulation of membrane raft components.

These data have led us to propose a molecular model for the development of AFT in which the ethanol sensitivity of ethanol target proteins, such as BK channels, can be modulated by changing the membrane milleu in which they reside. SWI/SNF regulation of factors that modify lipid rafts may be required if one of the mechanisms of AFT is for ethanol target proteins to be moved into or out of lipid rafts to normalize their function if they are exposed to ethanol. AFT in our system is observable within 30 min, and such modulation could be very quick, since it would not rely on transcription or changes in protein abundance. If there are appropriate lipid raft components available, then the system could be poised to respond to ethanol quickly when it is encountered.

### SWI/SNF acts through *cbp-1* to regulate AFT

Generally, gene families have expanded in more complex organisms, so our 603 candidate *C. elegans* genes are homologous to 874 human genes. As a first analysis of our dataset, we asked if any of the homologs of the genes in our candidate dataset have been implicated in alcohol response behaviors in other studies, by searching PubMed for coincidence of the human gene name and alcohol response search terms such as ‘alcohol dependence’ and ‘alcohol use disorder,’ and then further narrowed our search by filtering these by a mention of epigenetic regulation. This narrowed our list to 195 genes (Additional file 4). We noted that among the top 20 of these genes (Table 1), two, CREBBP (encoding CBP) and EP300 (encoding p300), are homologs of the same *C. elegans* gene, *cbp-1*. Both CBP and p300 have been shown to be involved in ethanol effects in several systems [28, 30, 42–44]. *cbp-1* is also interesting to us because CBP is known to act in concert with SWI/SNF complexes in epigenetic modulation of gene transcription [45, 46]. Together, these make *cbp-1* an excellent candidate mediator of the SWI/SNF effect on AFT. We tested strains heterozygous for two different loss of function mutations in *cbp-1* and found that *cbp-1/+* animals had defects in the development of AFT, demonstrating that this SWI/SNF target gene is important in this process. We favor a model in which SWI/SNF can modulate the transcription of *cbp-1*, and, CBP-1 and the SWI/SNF complex act in concert to regulate the transcription of effector genes that are required for the development of AFT. CBP-1 may also interact with other transcription factors to regulate the expression of effector genes. Intriguingly, both p300 and CBP have direct interactions with human CTBP family members in complex regulatory relationships [47–50]. CTBP1 and CTBP2 are transcriptional repressors that are homologous to *C. elegans ctbp-1*. We previously found that *ctbp-1* is required for normal AFT in worms [39], further suggesting a mechanism by which *cbp-1* may play a role in AFT. Our ultimate goal is to identify the direct effectors of AFT, to further our molecular understanding of this important aspect of the physiological effects of alcohol.

## Conclusions

This work describes the transcriptional targets of two subunits of the SWI/SNF chromatin remodeling complex in *C. elegans*. We generated one dataset of genes whose transcription is regulated by SWSN-9 in neurons. The second dataset is genes whose transcription requires SWSN-1 in adult tissues. Both of these genes are required for the development of acute functional tolerance, so we examined the intersection of these datasets, and identified 603 genes whose regulation is dependent on SWSN-1 in adults and SWSN-9 in neurons. Among these genes we expect to find the effectors of AFT. Pathway enrichment analysis identified genes involved in membrane rafts, suggesting that the regulation of membrane microarchitecture may play an important role in AFT. The candidate gene *cbp-1* is homologous to mammalian genes that have been implicated in ethanol respose behaviors, and we found that *cbp-1* function is required for normal AFT. This dataset provides a rich resource with which to examine the mechanisms underlying the development of acute functional tolerance to ethanol.

## Methods

### Strains

*C. elegans* strains were cultured as described previously [51, 52]. All strains were grown at 20 °C unless otherwise specified and were derived from the Bristol strain N2. Strains were obtained from the *Caenorhabditis* Genetics Center or were generated as described below. The lethal *cbp-1* alleles were maintained over *hT2[bli-4(e937) let-?(q782) qIs48],* which serves as a balancer chromosome for *LGI and LGIII.* The following alleles were used in this study and are described in *C. elegans II* [53], cited references, or this work:

*LGII: ttTi5605* [22], *rdIs52 [Pric-19::swsn-9::gfp; Cb-unc-119]* (this work), *rdIs49[Cb-unc-119]* (this work).

*LGIII: unc-119(ed9)* [54], *swsn-9(ok1354)* [55], *swsn-1(os22ts)* [15], *cbp-1(ok1491)* [56]*, cbp-1(bm2)* [57].

### *cbp-1* genetic analysis

Two *cbp-1* alleles were used in this study. Each allele was backcrossed at least six times to wild type (N2) worms before testing. Homozygous *cbp-1* loss-of-function alleles are lethal; therefore they were backcrossed alternately to N2 and a genetic balancer chromosome and they were tested as heterozygotes over a wild-type or balancer chromosome. To generate *cbp-1(lf)/+* worms, balanced strains were crossed with wild type males and *cbp-1/+* worms were identified in the F1 generation by the absence of a GFP marker on the balancer chromosome. During backcrossing, individuals were genotyped by PCR using primers flanking the mutations; deletions were detected by their shorter PCR product. All primers used in this study are listed in Additional file 5.

### Single copy neuronal rescue of *swsn-9(ok1354)*

We generated a single copy insertion of *Pric-19::swsn-9::GFP* using the MosSCI technique [22]. The repair plasmid was generated using Gibson assembly [58]. Briefly, the MosSCI repair plasmid pCFJ151 was digested with XhoI and SpeI and *Pric-19::swsn-9::GFP* was amplified from pRA570 [10] using primers that overlap with pCFJ151 on their 5′ ends. The two fragments were assembled using the NEBuilder High Fidelity Gibson Assembly master mix (NEB, Ipswich, MA) and the resulting plasmid was sequenced to ascertain that no mutations had been introduced during the cloning. Both the *Pric-19::swsn-9::GFP* repair plasmid (pRA590) and the empty repair plasmid (pCFJ151) were injected into EG4322 [*ttTi5605; unc-119(ed9)*] for insertion onto linkage group II. The resulting alleles, *rdIs52 [Pric-19::swsn-9::GFP; Cb-unc-119+]* and *rdIs49 [Cb-unc-119+],* were crossed into the *swsn-9(ok1354)* mutant background and the resulting strains, RA577 *swsn-9(ok1354); rdIs52 [Pric-19::swsn-9::gfp; Cb-unc-119]* and RA629 *swsn-9(ok1354); rdIs49[Cb-unc-119]*, were used for behavioral analysis and RNA sequencing*.*

### RNA sample collection

Five replicates were performed on different days; strains that were compared to one another were collected in parallel. We isolated mRNA from young adult worms in order to minimize embryonic transcripts in our samples. Wild-type and *swsn-1(os22ts)* strains (N2 and HS304) were reared at 15 °C through the fourth larval stage (L4) and then shifted to 25 °C; *swsn-9(ok1354)* strains (RA577 and RA629) were maintained at 20 °C continuously. Approximately 250 L4 worms were placed onto a plate containing OP50 and allowed to develop until most of the population was at the young adult stage and had not yet begun to lay eggs. At this time, any remaining L4 worms were removed from the plates and young adult worms were washed from the plate with M9 medium. Each replicate (*n* = 1) consisted of this pool of approximately 250 worms. The worms were rinsed once with M9 and stored in Trizol (Ambion, Carlsbad, CA) at − 80 °C until RNA preparation.

### RNA sequencing and analysis

Total RNA was isolated using the miRNeasy kit with on-column DNase I digestion (Qiagen, Venlo, Netherlands). RNA integrity was assessed on four representative samples using the Experion Automated Electrophoresis Station (Bio-Rad, Hercules, CA). All samples had RQI values ranging from 9.8 to 10.

Indexed sequencing libraries were prepared from polyA(+) selected RNA and sequenced at the Genomic Services Lab at Hudson Alpha (https://gsl.hudsonalpha.org/index). The libraries were sequenced as 50-base, paired-end reads, to an average read depth of 25 million reads per sample using an Illumina HiSeq v4 2500 (Illumina, San Diego, CA). We used FastQC (https://www.bioinformatics.babraham.ac.uk/projects/fastqc/) to examine the raw RNA sequencing data and found that they were of high quality and did not contain Illumina adapter sequences. Sequences were aligned to the *C. elegans* genome (Ensembl genome assembly release WBcel325) using Tophat2 version 2.1.1 [59], with Bowtie2 version 2.2.9 as the alignment algorithm. The GTF option was used to provide Tophat with a set of gene model annotations and the following parameters were specified (max-multihits 1, mate-inner-dist 200, −I 18000 –I 40). Aligned reads were sorted and indexed using SAMtools [60]. Gene-based read counts were obtained using HTSeq version 0.6.1 [61], using the Caenorhabditis_elegans.WBcel235.86.gtf annotation file with the union overlap resolution mode. Differential expression was determined using DESeq2 version 1.18.1 with alpha set to 0.05 [16]. FPKM (Fragments Per Kilobase of exon per Million fragments mapped) values were obtained using Cufflinks version 2.2.1 [62]. Mean FPKM values are reported, any values for which the FPKM status is FAIL were excluded. To examine the variance among our samples and replicates, we performed principal component analysis on regularized log transformed data using the rlogTransformation and plotPCA functions in DESeq2 [24]. Pearson’s correlation matrices were generated from read counts using iDEP [63] with no filtering for genes with minimum counts per million (CPM) and normalization using log_2_(CPM) (Additional file 5). Volcano plots were generated from read counts using iDEP [63] with parameters set to match our DESeq2 analysis (no CPM filtering, alpha = 0.05, fold change > 1). GO term enrichment for the differentially expressed genes (DEGs) was determined using the statistical overrepresentation test in PANTHER [64–66]. Gene lists were compared to all *C. elegans* genes in PANTHER using the GO biological process, cellular component, and molecular function datasets and Fisher’s exact test with false discovery rate (FDR) correction (FDR < 0.05). Overrepresented GO terms were clustered into hierarchical groups; only the most specific subclass is reported. k-means clustering of gene expression was performed using iDEP [63], with filtering to remove genes with fewer than 0.5 CPM in at least five samples. The top 2000 most variable genes were chosen based on the distribution of standard deviations for all genes and k was set (*k* = 7) such that it produced distinct and largely non-overlapping clusters when visualized using a t-distributed stochastic neighbour embedding (t-SNE) map [67].

### PubMed database searches

We created two web-based applications to facilitate searching the NCBI PubMed database with a large list of genes (www.geneinvestigator.com). The advantage of these applications is that they loop through the gene list, searching PubMed one gene at a time, thus providing search results on a gene by gene basis. They include a fully customizable search expression that is concatenated with the gene name using the Boolean operator AND. The search expression can include any number of terms linked by the Boolean operators AND, OR, and NOT. The ‘counts’ application returns a table with the number of citations matching each gene. The ‘retrieve’ application retrieves the twenty most recent citations for each gene.

### Locomotion assays

Locomotion tracking and analysis was performed as described previously [68, 69], with minor modifications. We treated the animals with 400 mM exogenous ethanol. The waxy cuticle of *C. elegans* is resistant to the passage of many chemicals, including ethanol [70, 71] so that in these experiments, the tissue concentration of ethanol is approximately 12% of the exogenous concentration, or approximately 48 mM [71]. Worms continue to accumulate ethanol over the course of the assay, so that there is a slightly higher concentration at 30 min relative to 10 min; behavioral improvement in this time is not due to pharmacokinetic effects, but rather to the development of acute functional tolerance to ethanol [71]. Ten first day adult worms were placed in copper rings that had been melted into the surface of agar plates containing 0 mM or 400 mM ethanol. Groups were matched so that worms from the same population were always treated with 0 mM and 400 mM ethanol in parallel. The locomotion of the worms was recorded for 2 min at 10 min of exposure and again at 30 min of exposure. The average speed was calculated for each group of worms (a treatment group of 10 worms yielded (*n* = 1)) using Image Pro Plus software (Media Cybernetics, Inc., Rockville, MD). Six trials were performed for each genotype. Relative speeds were calculated (treated speed/untreated speed × 100) for the 10 and 30 min exposure time points. Initial sensitivity to ethanol is measured by the relative speed of the worms after 10 min of exposure. Development of acute functional tolerance (AFT) was determined as a statistically significant difference between the relative speed of the animals after 10 min of ethanol exposure and the relative speed of the same animals after 30 min of ethanol exposure. One-tailed paired Student’s *t* tests were used for statistical comparisons of the relative speeds at the two time points. Comparisons of initial sensitivity and recovered speed across strains were made using 1-way ANOVA, with Tukey’s multiple comparison post-hoc test. Only animals that were tested simultaneously on the same plates were compared to each other.

## Supplementary information

**Additional file 1: **Differential gene expression analysis results. Included in this file are: 1- *swsn-1(os22ts)* DEGs identified by DESeq2 (FDR < 0.05 and fold-change ≥1). 2- *swsn-9(ok1354)* DEGs identified by DESeq2 (FDR < 0.05 and fold-change ≥1). 3- Intersection of *swsn-1(os22ts)* and *swsn-9(ok1354)* DEGs.

**Additional file 2 **GO term enrichment analysis of the differentially expressed genes. Included in this file are: 1- GO biological process, cellular component, and molecular function analysis of *swsn-1(os22ts)* DEGs (FDR < 0.05). 2- GO biological process, cellular component, and molecular function analysis of *swsn-9(ok1354)* DEGs (FDR < 0.05). 3- GO biological process, cellular component, and molecular function analysis of genes in the intersection of *swsn-1(os22ts)* and *swsn-9(ok1354)* DEGs (FDR < 0.05). 4- *swsn-1(os22ts)* and *swsn-9(ok1354)* (intersection) DEGs with the GO biological process term [GO:0045087] “innate immune response”. 5- *swsn-1(os22ts)* and *swsn-9(ok1354)* (intersection) DEGs with the GO cellular component term [GO:0045121] “membrane raft”.

**Additional file 3: **k-means clustering of wild type, *swsn-1,* and *swsn-9* gene expression data. Included in this file are: 1- Distribution of gene standard deviations across all three datsets. 2- k-means clustering of the top 2000 most variable genes (*k* = 7). 3- Results of DIOPT analysis for cluster C genes. 4- Results of DIOPT analysis for cluster F genes. 5- Cluster C and F genes compared with PBRM1 targets identified in [46]. 6- GO biological process terms that are overrepresented in each of the clusters. 7- GO cellular component terms that are overrepresented in each of the clusters. 8- GO molecular function terms that are overrepresented in each of the clusters.

**Additional file 4: **Analysis of human homologs of SWI/SNF-regulated genes. Included in this file are: 1- Results of DIOPT analysis for all genes in the intersection between *swsn-1(os22ts)* and *swsn-9(ok1354)* datasets. 2- Disease terms related to alcohol or substance use in the DisGeNET database. 3- Human homologs with gene disease associations (GDA) for alcohol/substance use terms on DisGeNET. 4- Citations on PubMed linking the human homologs to alcohol, alcohol-related diseases/diagnoses, and epigenetics. 5- Genes for which there are PubMed citations for alcohol phenotypes and epigenetics. 6- Top 20 genes from the PubMed analysis mapped back to *C. elegans* genes. 7- PubMed citations for the top 20 genes; limit of 20 citations returned.

**Additional file 5.** Supplemental information for Methods. Included in this file are: 1- Primers used in this study. 2- Total read counts for all RNA sequencing libraries. 3- Pearson’s correlation matrices for RNA sequencing samples and replicates.

## Data Availability

The RNA sequencing dataset generated during the current study is available in the NCBI SRA repository, accession number PRJNA611844, and the results are included with this article in tables and additional files.

## Abbreviations

AUD: Alcohol use disorder
LR: Level of response
AFT: Acute functional tolerance
BAF: BRG1- or BRM-associated factors
PBAF: Polybromo-associated BAF
SWI/SNF: SWItching defective/Sucrose Non-Fermenting chromatin remodeling complex
GFP: Green fluorescent protein
CPM: Counts per million
PCR: Polymerase chain reaction
DIOPT: DRSC Integrative Ortholog Prediction Tool
GO: Gene ontology
MosSCI: Mos-1 mediated single copy insertion
DEG: Differentially expressed gene
FPKM: Fragments Per Kilobase of exon per Million fragments mapped
FDR: False discovery rate

## References

[1] Surveilance Report #113: Apparent per Capita Alcohol Consumption: National, State, and Regional Trends, 1977–2017 [https://pubs.niaaa.nih.gov/publications/surveillance113/CONS17.htm]. Accessed 23 Mar 2020.

[2] White AM, Castle IP, Hingson RW, Powell PA (2020). Using death certificates to explore changes in alcohol-related mortality in the United States, 1999 to 2017. Alcohol Clin Exp Res.

[3] Prescott CA, Kendler KS (1999). Genetic and environmental contributions to alcohol abuse and dependence in a population-based sample of male twins. Am J Psychiatry.

[4] Edwards AC, Deak JD, Gizer IR, Lai D, Chatzinakos C, Wilhelmsen KP, Lindsay J, Heron J, Hickman M, Webb BT (2018). Meta-analysis of genetic influences on initial alcohol sensitivity. Alcohol Clin Exp Res.

[5] Heath AC, Madden PA, Bucholz KK, Dinwiddie SH, Slutske WS, Bierut LJ, Rohrbaugh JW, Statham DJ, Dunne MP, Whitfield JB (1999). Genetic differences in alcohol sensitivity and the inheritance of alcoholism risk. Psychol Med.

[6] Schuckit MA (2018). A critical review of methods and results in the search for genetic contributors to alcohol sensitivity. Alcohol Clin Exp Res.

[7] Schuckit MA (2000). Genetics of the risk for alcoholism. Am J Addict.

[8] Schuckit MA (1994). Low level of response to alcohol as a predictor of future alcoholism. Am J Psychiatr.

[9] Kalu N, Ramchandani VA, Marshall V, Scott D, Ferguson C, Cain G, Taylor R (2012). Heritability of level of response and association with recent drinking history in nonalcohol-dependent drinkers. Alcohol Clin Exp Res.

[10] Mathies LD, Blackwell GG, Austin MK, Edwards AC, Riley BP, Davies AG, Bettinger JC (2015). SWI/SNF chromatin remodeling regulates alcohol response behaviors in *Caenorhabditis elegans* and is associated with alcohol dependence in humans. Proc Natl Acad Sci U S A.

[11] Clapier CR, Cairns BR (2009). The biology of chromatin remodeling complexes. Annu Rev Biochem.

[12] Wu JI, Lessard J, Crabtree GR (2009). Understanding the words of chromatin regulation. Cell.

[13] Kadoch C, Crabtree GR (2015). Mammalian SWI/SNF chromatin remodeling complexes and cancer: mechanistic insights gained from human genomics. Sci Adv.

[14] Mathies LD, Aliev F, Investigators C, Davies AG, Dick DM, Bettinger JC (2017). Variation in SWI/SNF chromatin remodeling complex proteins is associated with alcohol dependence and antisocial behavior in human populations. Alcohol Clin Exp Res.

[15] Sawa H, Kouike H, Okano H (2000). Components of the SWI/SNF complex are required for asymmetric cell division in *C. elegans*. Mol Cell.

[16] Love MI, Huber W, Anders S (2014). Moderated estimation of fold change and dispersion for RNA-seq data with DESeq2. Genome Biol.

[17] Jordan-Pla A, Yu S, Waldholm J, Kallman T, Ostlund Farrants AK, Visa N (2018). SWI/SNF regulates half of its targets without the need of ATP-driven nucleosome remodeling by Brahma. BMC Genomics.

[18] Riedel CG, Dowen RH, Lourenco GF, Kirienko NV, Heimbucher T, West JA, Bowman SK, Kingston RE, Dillin A, Asara JM (2013). DAF-16 employs the chromatin remodeller SWI/SNF to promote stress resistance and longevity. Nat Cell Biol.

[19] Euskirchen GM, Auerbach RK, Davidov E, Gianoulis TA, Zhong G, Rozowsky J, Bhardwaj N, Gerstein MB, Snyder M (2011). Diverse roles and interactions of the SWI/SNF chromatin remodeling complex revealed using global approaches. PLoS Genet.

[20] Attanasio C, Nord AS, Zhu Y, Blow MJ, Biddie SC, Mendenhall EM, Dixon J, Wright C, Hosseini R, Akiyama JA (2014). Tissue-specific SMARCA4 binding at active and repressed regulatory elements during embryogenesis. Genome Res.

[21] Mello CC, Kramer JM, Stinchcomb D, Ambros V (1991). Efficient gene transfer in *C. elegans*: extrachromosomal maintenance and integration of transforming sequences. EMBO J.

[22] Frokjaer-Jensen C, Davis MW, Hopkins CE, Newman BJ, Thummel JM, Olesen SP, Grunnet M, Jorgensen EM (2008). Single-copy insertion of transgenes in *Caenorhabditis elegans*. Nat Genet.

[23] Kuzmanov A, Karina EI, Kirienko NV, Fay DS (2014). The conserved PBAF nucleosome-remodeling complex mediates the response to stress in *Caenorhabditis elegans*. Mol Cell Biol.

[24] Chowdhury B, Porter EG, Stewart JC, Ferreira CR, Schipma MJ, Dykhuizen EC (2016). PBRM1 regulates the expression of genes involved in metabolism and cell adhesion in renal clear cell carcinoma. PLoS One.

[25] Hu Y, Flockhart I, Vinayagam A, Bergwitz C, Berger B, Perrimon N, Mohr SE (2011). An integrative approach to ortholog prediction for disease-focused and other functional studies. BMC Bioinformatics.

[26] Pinero J, Ramirez-Anguita JM, Sauch-Pitarch J, Ronzano F, Centeno E, Sanz F, Furlong LI (2020). The DisGeNET knowledge platform for disease genomics: 2019 update. Nucleic Acids Res.

[27] Ghezzi A, Li X, Lew LK, Wijesekera TP, Atkinson NS (2017). Alcohol-induced Neuroadaptation is orchestrated by the histone Acetyltransferase CBP. Front Mol Neurosci.

[28] Pandey SC, Kyzar EJ, Zhang H (2017). Epigenetic basis of the dark side of alcohol addiction. Neuropharmacology.

[29] Pandey SC, Ugale R, Zhang H, Tang L, Prakash A (2008). Brain chromatin remodeling: a novel mechanism of alcoholism. J Neurosci.

[30] Zhang H, Kyzar EJ, Bohnsack JP, Kokare DM, Teppen T, Pandey SC (2018). Adolescent alcohol exposure epigenetically regulates CREB signaling in the adult amygdala. Sci Rep.

[31] Dulman RS, Auta J, Teppen T, Pandey SC (2019). Acute ethanol produces Ataxia and induces Fmr1 expression via histone modifications in the rat cerebellum. Alcohol Clin Exp Res.

[32] Kumar D, Deb I, Chakraborty J, Mukhopadhyay S, Das S (2011). A polymorphism of the CREB binding protein (CREBBP) gene is a risk factor for addiction. Brain Res.

[33] Dopico AM, Bukiya AN, Bettinger JC (2018). Voltage-sensitive potassium channels of the BK type and their coding genes are alcohol targets in neurons. Handb Exp Pharmacol.

[34] Bukiya AN, Dopico AM (2019). Regulation of BK Channel activity by cholesterol and its derivatives. Adv Exp Med Biol.

[35] Dopico AM, Bukiya AN, Kuntamallappanavar G, Liu J (2016). Modulation of BK channels by ethanol. Int Rev Neurobiol.

[36] Davies AG, Pierce-Shimomura JT, Kim H, VanHoven MK, Thiele TR, Bonci A, Bargmann CI, McIntire SL (2003). A central role of the BK potassium channel in behavioral responses to ethanol in *C. elegans*. Cell.

[37] Simons K, Toomre D (2000). Lipid rafts and signal transduction. Nat Rev Mol Cell Biol.

[38] Lucero HA, Robbins PW (2004). Lipid rafts-protein association and the regulation of protein activity. Arch Biochem Biophys.

[39] Bettinger JC, Leung K, Bolling MH, Goldsmith AD, Davies AG (2012). Lipid environment modulates the development of acute tolerance to ethanol in *Caenorhabditis elegans*. PLoS One.

[40] Schley PD, Brindley DN, Field CJ (2007). (n-3) PUFA alter raft lipid composition and decrease epidermal growth factor receptor levels in lipid rafts of human breast cancer cells. J Nutr.

[41] Hellwing C, Tigistu-Sahle F, Fuhrmann H, Kakela R, Schumann J (2018). Lipid composition of membrane microdomains isolated detergent-free from PUFA supplemented RAW264.7 macrophages. J Cell Physiol.

[42] Donde H, Ghare S, Joshi-Barve S, Zhang J, Vadhanam MV, Gobejishvili L, Lorkiewicz P, Srivastava S, McClain CJ, Barve S. Tributyrin inhibits ethanol-induced epigenetic repression of CPT-1A and attenuates hepatic Steatosis and injury. Cell Mol Gastroenterol Hepatol. 2019. 10.1016/j.jcmgh.2019.10.005 PMC7078548 31654770

[43] Wu Z, Pan Z, Wen Y, Xiao H, Shangguan Y, Wang H, Chen L (2020). Egr1/p300/ACE signal mediates postnatal osteopenia in female rat offspring induced by prenatal ethanol exposure. Food Chem Toxicol.

[44] Teppen TL, Krishnan HR, Zhang H, Sakharkar AJ, Pandey SC (2016). The potential role of Amygdaloid MicroRNA-494 in alcohol-induced Anxiolysis. Biol Psychiatry.

[45] Alver BH, Kim KH, Lu P, Wang X, Manchester HE, Wang W, Haswell JR, Park PJ, Roberts CW (2017). The SWI/SNF chromatin remodelling complex is required for maintenance of lineage specific enhancers. Nat Commun.

[46] Huang ZQ, Li J, Sachs LM, Cole PA, Wong J (2003). A role for cofactor-cofactor and cofactor-histone interactions in targeting p300, SWI/SNF and mediator for transcription. EMBO J.

[47] Kim JH, Cho EJ, Kim ST, Youn HD (2005). CtBP represses p300-mediated transcriptional activation by direct association with its bromodomain. Nat Struct Mol Biol.

[48] Senyuk V, Sinha KK, Nucifora G (2005). Corepressor CtBP1 interacts with and specifically inhibits CBP activity. Arch Biochem Biophys.

[49] Zhao LJ, Subramanian T, Zhou Y, Chinnadurai G (2006). Acetylation by p300 regulates nuclear localization and function of the transcriptional corepressor CtBP2. J Biol Chem.

[50] Zhang W, Duan N, Zhang Q, Song T, Li Z, Chen X, Wang K (2018). The intracellular NADH level regulates atrophic nonunion pathogenesis through the CtBP2-p300-Runx2 transcriptional complex. Int J Biol Sci.

[51] Wood WB, Wood WB (1988). Introduction to *C. elegans* Biology. The Nematode *Caenorhabditis elegans*.

[52] Brenner S (1974). The genetics of *Caenorhabditis elegans*. Genetics.

[53] Hodgkin J, Riddle DL, Blumenthal T, Meyer BJ, Priess JR (1997). Appendix 1. Genetics. *C elegans* II.

[54] Maduro M, Pilgrim D (1995). Identification and cloning of *unc-119*, a gene expressed in the *Caenorhabditis elegans* nervous system. Genetics.

[55] Large EE, Mathies LD (2014). *Caenorhabditis elegans* SWI/SNF subunits control sequential developmental stages in the somatic gonad. G3 (Bethesda).

[56] The *C. elegans* Deletion Mutant Consortium. Large-scale screening for targeted knockouts in the *Caenorhabditis elegans* genome. G3 (Bethesda) 2012;2(11):1415–25. 10.1534/g3.112.003830 PMC3484672 23173093

[57] Victor M, Bei Y, Gay F, Calvo D, Mello C, Shi Y (2002). HAT activity is essential for CBP-1-dependent transcription and differentiation in *Caenorhabditis elegans*. EMBO Rep.

[58] Gibson DG, Young L, Chuang RY, Venter JC, Hutchison CA, Smith HO (2009). Enzymatic assembly of DNA molecules up to several hundred kilobases. Nat Methods.

[59] Kim D, Pertea G, Trapnell C, Pimentel H, Kelley R, Salzberg SL (2013). TopHat2: accurate alignment of transcriptomes in the presence of insertions, deletions and gene fusions. Genome Biol.

[60] Li H, Handsaker B, Wysoker A, Fennell T, Ruan J, Homer N, Marth G, Abecasis G, Durbin R (2009). Genome project data processing S. The sequence alignment/map format and SAMtools. Bioinformatics.

[61] Anders S, Pyl PT, Huber W (2015). HTSeq--a Python framework to work with high-throughput sequencing data. Bioinformatics.

[62] Trapnell C, Williams BA, Pertea G, Mortazavi A, Kwan G, van Baren MJ, Salzberg SL, Wold BJ, Pachter L (2010). Transcript assembly and quantification by RNA-Seq reveals unannotated transcripts and isoform switching during cell differentiation. Nat Biotechnol.

[63] Ge SX, Son EW, Yao R (2018). iDEP: an integrated web application for differential expression and pathway analysis of RNA-Seq data. BMC Bioinformatics.

[64] Thomas PD, Kejariwal A, Campbell MJ, Mi H, Diemer K, Guo N, Ladunga I, Ulitsky-Lazareva B, Muruganujan A, Rabkin S (2003). PANTHER: a browsable database of gene products organized by biological function, using curated protein family and subfamily classification. Nucleic Acids Res.

[65] Mi H, Muruganujan A, Casagrande JT, Thomas PD (2013). Large-scale gene function analysis with the PANTHER classification system. Nat Protoc.

[66] Mi H, Huang X, Muruganujan A, Tang H, Mills C, Kang D, Thomas PD (2017). PANTHER version 11: expanded annotation data from gene ontology and Reactome pathways, and data analysis tool enhancements. Nucleic Acids Res.

[67] van der Maaten LJP, Hinton GE (2008). Visualizing High-Dimensional Data Using t-SNE. J Mach Learn Res.

[68] Davies AG, Bettinger JC, Thiele TR, Judy ME, McIntire SL (2004). Natural variation in the *npr-1* gene modifies ethanol responses of wild strains of *C. elegans*. Neuron.

[69] Davies AG, Blackwell GG, Raabe RC, Bettinger JC. An assay for measuring the effects of ethanol on the locomotion speed of *Caenorhabditis elegans*. J Vis Exp. 2015;98. 10.3791/52681 PMC4476067 25938273

[70] Burns AR, Wallace IM, Wildenhain J, Tyers M, Giaever G, Bader GD, Nislow C, Cutler SR, Roy PJ (2010). A predictive model for drug bioaccumulation and bioactivity in *Caenorhabditis elegans*. Nat Chem Biol.

[71] Alaimo J, Davis S, Song S, Burnette C, Grotewiel M, Shelton K, Pierce-Shimomura J, Davies A, Bettinger J (2012). Ethanol metabolism and osmolarity modify behavioral responses to ethanol in *C. elegans*. Alcohol Clin Exp Res.

